# Cardiac involvement in Anderson–Fabry disease. The role of advanced echocardiography

**DOI:** 10.3389/fcvm.2024.1440636

**Published:** 2024-09-02

**Authors:** Letizia Spinelli, Antonio Bianco, Eleonora Riccio, Antonio Pisani, Guido Iaccarino

**Affiliations:** ^1^ Interdepartmental Research Center for Hypertension and Related Conditions, University of Naples, Naples, Italy; ^2^ Department of Public Health, Federico II University, Naples, Italy; ^3^ Department of Clinical Medicine and Surgery, Federico II University, Naples, Italy

**Keywords:** Anderson–Fabry disease, cardiac function, myocardial strain, speckle-tracking echocardiography, tissue doppler imaging

## Abstract

Anderson–Fabry disease (AFD) is a lysosomal storage disorder, depending on defects in alpha galactosidase A activity, due to a mutation in the galactosidase alpha gene. Cardiovascular involvement represents the leading cause of death in AFD. Cardiac imaging plays a key role in the evaluation and management of AFD patients. Echocardiography is the first-line imaging modality for the identification of the typical features of AFD cardiomyopathy. Advanced echocardiography that allows assessment of myocardial deformation has provided insights into the cardiac functional status of AFD patients. The present review highlights the value and the perspectives of advanced ultrasound imaging in AFD.

## Introduction

Anderson–Fabry disease (AFD) is a lipidosis caused by deficient αGLA (α-galactosidase A) enzyme activity due to a mutation in the galactosidase alpha gene leading to progressive lysosomal accumulation of complex sphingolipids in vascular endothelial and smooth-muscle cells throughout the body and in the cells of kidney, nervous system, eyes, and heart (1, 2). Cardiac involvement represents the main cause of impaired quality of life and of reduced life expectancy (3). In the heart, accumulation of globotriaosylceramide (Gb3) affects all cell types, including myocytes, endocardium, valvular fibroblasts, and specific myocardium cardiomyocytes. Imaging represents a key tool in the diagnostic and therapeutic approaches to AFD cardiac manifestations (4, 5). Two-dimensional (2D) transthoracic echocardiographic assessment is the first step in to detail morphologic and functional aspects of heart in AFD, namely: left ventricular (LV) concentric hypertrophy, preserved ejection fraction, disproportionate hypertrophy of papillary muscles and, often, right ventricular (RV) hypertrophy. Advancement in cardiac ultrasound imaging allows to quantify myocardial deformation in the different spatial directions offering an innovative evaluation of LV function. Tissue Doppler strain rate curves and speckle tracking echocardiography (STE) unrevealed an impairment of myocardial function in AFD patients with preserved ejection fraction. Given its angle dependency, tissue Doppler is limited in assessing LV apex. Owing the ability to assess myocardial deformation in all segments of LV walls, STE allows to overcome such limitations. Myocardial deformation measurements by 2D STE have been validated against both sonomicrometry and 2D-tagged cardiac magnetic resonance imaging (6). Further technological advancement of real-time three-dimensional (3D) echocardiography has developed software that tracks the motion of speckles irrespective of their direction and allows to obtain a homogeneous spatial distribution of all three components of the myocardial displacement vector. Myocardial strain can be analyzed from full-volume acquisitions, potentially overcoming the out-of-plane loss of speckles associated with 2D STE analyses. Thus, a series of studies revealing the impairment of LV function caused by AFD flourished during the last two decades. New insights have been provided in subclinical detection of AFD-related abnormalities as well as in disease staging and in prognostication. Given that AFD is a rare disease, most studies offered insights into small cohorts of patients. This paper aims to provide a comprehensive review of current knowledge and of ongoing research into the evaluation of AFD cardiomyopathy with use of advanced echocardiography.

## Left ventricular systolic function

The heart in AFD patients presents a phenocopy of hypertrophic cardiomyopathy with preserved LV ejection until the late stages of disease. Strain imaging revealed that the AFD patients may have an impairment of LV systolic function, despite an ejection fraction within the normal range. Studies using tissue Doppler echocardiography demonstrated subclinical LV dysfunction even in early stages of disease (7, 8). Weidemann et al. found out that both peak systolic strain rate and systolic strain were significantly reduced in either the radial or longitudinal direction in 16 AFD patients compared with controls (9). In 2007, the same group described a double peak sign in tissue Doppler strain rate curves in myocardial segments with late gadolinium enhancement by magnetic resonance imaging (10) and demonstrated that a pattern-based analysis was more sensitive and more specific for detecting fibrosis than peak strain (11). Studies by STE showed a decrease in LV longitudinal strain (12–14) involving mainly basal segments (15–17) although apical segments were not completely spared, unlike amyloidosis related cardiomyopathy (15, 18). Moreover, AFD patients with LV hypertrophy were found to have a worse longitudinal function than patients with non-obstructive hypertrophic cardiomyopathy (14). By using the quantitative measurement of myocardial fibrosis with magnetic resonance imaging, Kramer et al, demonstrated an association between the impairment of longitudinal strain and the amount of myocardial replacement fibrosis (19). Interestingly, measuring time-to-peak longitudinal strain unveiled a high prevalence of intraventricular dyssynchrony in AFD patients with LV hypertrophy (20). Cardiac sympathetic denervation has been described in AFD related cardiomyopathy (21–24). It has been found that the presence of denervated areas affects segmental longitudinal strain yielding reduction of global LV function (25). Several studies have highlighted the reduction of LV global longitudinal function before the occurrence of LV hypertrophy, suggesting that myocardial functional impairment is an intrinsic feature of disease and not a consequence of increased LV mass (13, 17, 26, 27). In AFD patients, cardiomyocyte glycosphingolipid storage causes myofibrillolysis and myofilament derangement resulting in a detrimental functional effect (28). A study including a quite large cohort of patients with late- onset cardiac variant showed that AFD patients without LV hypertrophy still had a reduced global longitudinal strain when compared to healthy subjects, despite similar LV mass and morphology (29). It has been suggested that basal longitudinal strain should be considered when screening for cardiac involvement in AFD, particularly in female AFD patients with normal LV wall thickness (17). Reduction in longitudinal strain was found associated with low native T1 in AFD patients without LV hypertrophy (30, 31). Furthermore, in females carrying α-Gal A mutation and without LV hypertrophy, LV global longitudinal strain was impaired in presence of focal myocardial inflammation, identified as focal ^18^F-luorodeoxyglucose uptake by cardiac positron emission tomography (32).

There are limited data on the impairment of LV circumferential strain in AFD (13, 14, 19, 33). Circumferential strain refers to mid-wall fibers, the same myocardial portion where fibrosis finds its most typical distribution in AFD. Shanks et al. did not find any difference in circumferential function between AFD patients and age- and gender matched healthy subjects (13), while other studies by echocardiography (14, 21, 33) or cardiac magnetic resonance (34) demonstrated that, alongside with impairment in longitudinal function, AFD patients experienced the decrease of global circumferential strain and the loss of base to apex gradient irrespectively of LV geometry (14). Conversely, patients with nonobstructive hypertrophic cardiomyopathy compensated the decrease in longitudinal function with an increase in global circumferential strain and preservation of the base-to-apex gradient (14). Thus, the loss of base to apex gradient seems to be specific to AFD cardiomyopathy and could be caused by the greater impairment of subepicardial fibers, which are mainly responsible for circumferential strain (35).

**Figure 1:**
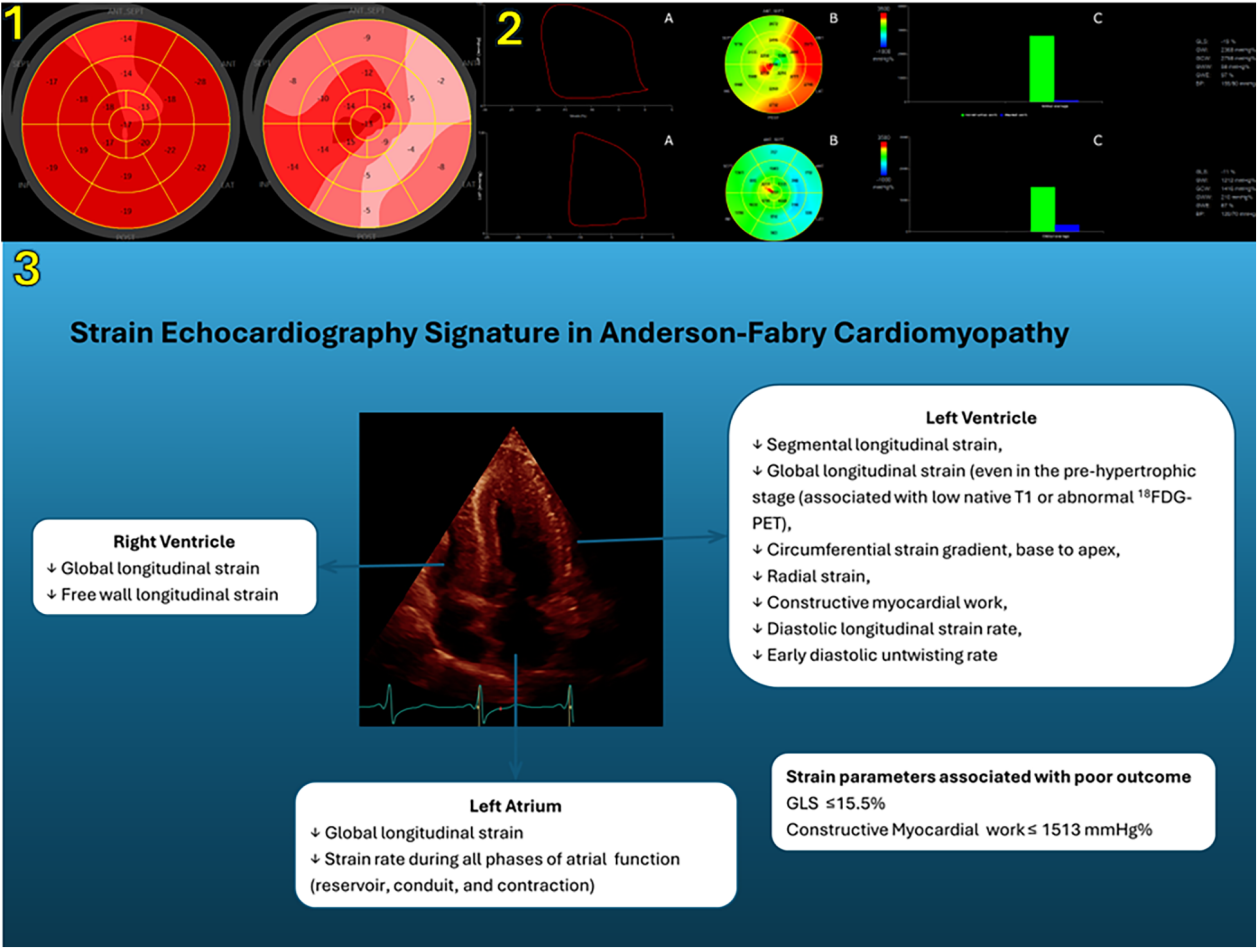
Panel 1. Left ventricular longitudinal strain measurements by means of two-dimensional speckle tracking echocardiography. Bull’s-eye rendering of LV longitudinal strain from a 42-year-old female AFD patient without LV hypertrophy (left) and a 53-year-old male AFD patient with LV hypertrophy (right). Panel 2. (**A**) Representative example of pressure–strain loops by estimated LV pressure and LV longitudinal strain; (**B**) Bull’s eye plot showing segmental LV myocardial work index in a 17-segment model; (**C**) constructive work (green column) and wasted work (blue column) values from the same patients as in the panel 1 (*upper*, female patient without LV hypertrophy; *lower*, male patient with LV hypertrophy). Panel 3 shows the role of strain echocardiography in the characterization of cardiac involvement in AFD. LV, left ventricular; AFD, Anderson–Fabry disease; SEPT, septal; LAT, lateral; ANT, anterior; INF, inferior; POST, posterior; LVP, left ventricular pressure; GLS, global longitudinal strain; GWI, global work index; GCW, global constructive work; GWW, global wasted work; GWE, global work efficiency; BP, blood pressure; ^18^F-FDG PET, ^18^F-luorodeoxyglucose uptake by cardiac positron emission tomography.

The data on LV radial strain are even more scarce. Color Doppler myocardial imaging demonstrated an impairment in radial strain rate of posterior wall in AFD patients with LV hypertrophy as well as in female patients with normal LV mass and evidence of late gadolinium enhancement by cardiac magnetic resonance (9, 36). Studies by 2D STE reported conflicting findings (13, 37). While an early study showed normal values of radial strain (13), another study including a larger population demonstrated an early deterioration in LV radial strain, affecting even patients without clear-cut wall hypertrophy (37). Interestingly, global longitudinal strain was significantly associated to LV mass whereas, radial strain was not. However, a recent study by 3D echocardiography has shown an inverse correlation between LV mass and radial strain in 75 AFD patients (51% with LV hypertrophy or concentric remodeling). The use in 3D analysis of a different method for radial strain assessment that was based on volume conservation might account for the different results (38). However, among the various myocardial deformation components, global longitudinal strain has shown the best ability in detecting subclinical LV systolic dysfunction. Figure 1 shows representative examples of longitudinal strain in AFD patients (panel 1). Nevertheless, longitudinal strain is influenced by loading conditions. Myocardial work derived by pressure-strain analysis is a novel non-invasive method to characterize myocardial deformation in relation to afterload conditions (39). Early findings indicate that myocardial work may have an additive value in the functional assessment of AFD cardiomyopathy (40, 41). In the Figure 1, representative examples of myocardial work from AFD patients are shown (panel 2).

## Left ventricular diastolic function

Progressive LV hypertrophy with preserved ejection fraction and diastolic dysfunction have been described as the major echocardiographic features of AFD cardiomyopathy (42). There is a growing awareness of diastolic dysfunction being an early sign of cardiac involvement in AFD. It has been suggested that the tissue Doppler derived diastolic index, namely early diastolic mitral annulus velocities (i.e., e′) could provide satisfactory preclinical evidence for diastolic dysfunction in patients with AFD (7). Yet, this was not found in subsequent studies (8, 29). The diastolic strain rate measured by 2D STE emerged as a sensitive tool in detecting diastolic dysfunction, better than conventional diastolic indices (43). Shanks et al. showed that longitudinal strain rate parameters, particularly those measured during early diastole, identify AFD patients from healthy controls, independent of LV hypertrophy and in a more specific manner than tissue Doppler measurements (13). A recent study confirmed the impairment in diastolic longitudinal strain rate of AFD patients without clear-cut LV wall hypertrophy (44). LV diastolic longitudinal strain rate is attenuated by myocardial fibrosis, a typical feature of AFD cardiomyopathy (45). Similarly, LV diastolic rotational mechanics may be also impaired in AFD patients. Indeed, reduced early diastole untwisting rate has been demonstrated associated to myocardial sympathetic denervation (21).

## Left atrial function

Histopathological findings demonstrated the accumulation of Gb3 in the left atrium (LA) of AFD patients supporting atrial myopathy (42, 46, 47). However, few studies have investigated the effects of AFD on LA size and mechanical function. LA acts as a blood reservoir during ventricular systole, as a passive conduit during the passage of blood from the pulmonary veins to the left ventricle during early diastole and as a contractile chamber to increase ventricular filling during atrial systole. Strain and strain rate imaging allows to assess atrial function via the analysis of the cardiac cycle. Boyd et al. used tissue Doppler imaging with a four-point segmental approach to assess LA strain and strain rate and demonstrated that LA systolic strain and early diastolic strain rate were selectively reduced in AFD patients with LV hypertrophy. Interestingly, LA enlargement and reduced atrial compliance were found in the subgroup without LV hypertrophy, despite a normal diastolic function with e′ values like those in controls (48). Almost all studies explored LA function using 2D STE, as the technique allows a complete assessment of endocardial strain. Morris et al. could detect LA myocardial dysfunction in AFD patients, even when LA volume was normal. However, in their study data on conduit function were not reported (12). Notwithstanding, a retrospective study comparing 50 AFD patients with 50 healthy control subjects demonstrated that LA reservoir, conduit, and contractile functions were all affected in AFD patients (49). Saccheri et al. analyzed LA function in AFD patients with LV hypertrophy in comparison with patients with hypertrophic cardiomyopathy and found out that both disorders exhibited a severe functional impairment, although LA volume was lower in AFD (50). Conversely, a lower LA volume and a lower impairment of all three phases of LA mechanics have been detected in AFD patients than in patients with cardiac amyloidosis (51, 52). Several data suggest that differential echocardiographic diagnostic work-up of unclear LV hypertrophy can be improved by integrating LA strain analysis. Frumkin et al. analyzed patients with AFD cardiomyopathy and patients with LV hypertrophy due to other causes and found that LA conduit strain showed the highest diagnostic accuracy to discriminate AFD, superior to the posterolateral strain impairment and papillary muscle hypertrophy pattern (53). Likewise, in a recent study by cardiac magnetic resonance imaging the impairment in LA reservoir strain performed better than the established approach using LV mass index and low native T1 in identifying early disease (54). Although none of the above parameters has so far been validated as independent predictor in large enough cohorts, deformation analysis by means of advanced echocardiography or other cardiac imaging modality has the potential to provide valuable insights into LA functional status of AFD patients. Bradyarrhythmia are common manifestations of cardiac involvement in AFD, often requiring pacemaker implantation. It has been demonstrated that LA reservoir dysfunction can be a useful marker associated to bradyarrhythmia (55). Atrial fibrillation is a possible complication of AFD occurring in about 13% of patients. The risk factors for atrial fibrillation hitherto identified are limited to age, LV hypertrophy and atrial dilatation. Few data suggest an association between the impairment of LA strain parameters with the occurrence of atrial fibrillation and stroke in FD patients (49). However, the role of LA dysfunction as a risk factor for atrial fibrillation needs to be addressed in large studies. Quite common features of central nervous system involvement in AFD are non-specific periventricular and deep white matter lesions along with silent lacunar infarctions of the brain. In a small cohort of AFD patients, Esposito et al. found that LA function expressed as peak atrial longitudinal strain was inversely associated with the presence of non-specific white matter lesions (56).

## Right ventricular function

Anatomopathological findings demonstrated that structural changes such as the accumulation of Gb3 take place also in the right ventricle (RV). RV hypertrophy, defined as wall thickness >5 mm, is more frequent with increasing age, and the extent is correlated with the degree of coexisting LV hypertrophy (57–61). When assessed by conventional echocardiography, indices of RV systolic function may be found within the normal range, even when severe RV hypertrophy is present (12, 60). Indeed, only in the late disease stages RV involvement progresses to severe systolic and diastolic RV dysfunction (57). Morris et al. (12) evaluated longitudinal systolic strain peak from the free and septal wall (i.e., RV strain) and just from the free wall of the RV (i.e., RV FW strain) and unveiled systolic dysfunction in 20% of patients (61, 62). Lillo found out that the physiologic difference between the RV-FW strain and the global RV strain was preserved regardless of the presence of overt cardiomyopathy (62). Compared to patients with hypertrophic cardiomyopathy, AFD patients showed worse RV FW longitudinal strain, despite comparable conventional parameters (52, 61). Conversely, a minimal involvement of RV function has been documented in AFD compared to cardiac amyloidosis (51). According to 2D echocardiography, RV involvement seems to be a late phenomenon of the disease as RV strain is preserved in the pre-hypertrophic phase (62). Nevertheless, a pilot study by 3D STE showed an early subclinical functional damage (63). Nevertheless, the putative 3D imaging advantages that derive from the independency from the through-plane phenomenon and the ability to provide real information on volume and wall deformation with no need for geometric assumptions (64), still need to be confirmed in larger patient cohorts.

## Advanced echocardiography and prognosis

Identifying patients who are at risk of adverse cardiac outcome may facilitate more evidence-based treatment guidance (65–67). The assessment of LV function by longitudinal strain has become widely adopted, but its prognostic value in AFD remains unclear. Early findings indicated a link between alterations in LV global longitudinal function and symptomatic status and prognosis (12). Interestingly, also basal longitudinal strain reduction was found associated with major adverse cardiovascular events (17). In a cohort of 96 AFD patients, global longitudinal strain showed an incremental prognostic value over clinical factors, LV mass index and diastolic function, during a median follow-up of 5.2 years (40). The prognostic value of LV global longitudinal strain has been confirmed by other studies including one by 3D echocardiography (38, 68, 69). Mechanical dispersion STE has been proposed as an additional risk marker (70). The only study utilizing strain derived myocardial work for the assessment of LV function in AFD suggested a higher accuracy of myocardial work in comparison with global longitudinal strain (GLS) in predicting event free survival has been observed, with constructive myocardial work being the best performing index (40).

## Advanced echocardiography and the effects of therapy

While there is a growing acceptance of the role of strain imaging in early detecting of cardiac involvement of AFD and thus, in determining the candidacy to disease- specific therapy, there is a lack of findings regarding its usefulness in assessing the effects of therapy Already in 2003, Weidemann et al. demonstrated a significant improvement in longitudinal and radial strain values by tissue Doppler after 1 year of enzyme replacement therapy (9), whereas the presence of myocardial fibrosis did not benefit from therapy over a period of 3 years (71). A significant decrease of longitudinal systolic strain rate at basal-mid level of LV lateral wall was observed in AFD patients treated prospectively with enzyme replacement therapy for 6 years (72). There are findings suggesting that enzyme replacement therapy may delay the onset of cardiac involvement, thus, supporting the initiation of therapy at an earlier stage of the disease (73). Recently, a significant improvement in apical circumferential strain was observed during enzyme replacement therapy (44) The effects of therapy on LA function have been scarcely investigated. Pichette et al. reported an improvement in LA reservoir strain and in some cases in conduit and contractile strains after 12 months of treatment (49). However, therapy was able to improve LA strain, but not to reduce LA volume (62). A recent study demonstrated no improvement, rather a stabilized LA strain in patients treated with enzyme replacement therapy as well as in those receiving chaperone therapy (74). Finally, therapy seems to have no direct impact on RV morphology and function (59).

## Advanced echocardiography: benefits and pitfalls

The diagnosis AFD is based on signs and symptoms suggestive of a systemic disease, family history, an absent or reduced (<5% of normal) leukocyte *α*-GalA activity level (in men) and is confirmed by genotype testing. Standard and advanced echocardiography are not enough to confirm diagnosis of AFD, but provide essential insights in the functional evaluation AFD, unrevealing myocardial dysfunction in patients with LV hypertrophy and preserved ejection fraction. Once a diagnosis of AFD disease has been established, the presence of abnormal global or segmental strain in an otherwise normal heart may be suggestive of early involvement and should trigger closer clinical follow-up. In the Figure 1, the role of strain echocardiography in identifying the features of heart involvement in AFD is shown (panel 3). When comparing standard and advanced echocardiography to other morphological analysis such as cardiac magnetic resonance, it has to be kept in mind that the information is often additive, more than alternative. Indeed, cardiac magnetic resonance can provide a precise evaluation of heart morphology and tissue characteristics. Native T1 mapping allows early detection of cardiac involvement in a pre-hypertrophic stage and can discriminate between control subjects and AFD patients without LV hypertrophy. Moreover, low myocardial T1 values in pre- hypertrophic stage correlate with reduced global longitudinal strain (30). However, its wide adoption is hampered by the lack of standardized cut-off values for T1 mapping as the analysis is influenced by imaging equipment and protocols. In this perspective, echocardiography has the advantage of being widely spread, less expensive and easily repeatable. Nevertheless, cardiac imaging findings, either by advanced echocardiography or by cardiac magnetic resonance, are not specific nor pathognomonic of AFD. Some feature can help diagnosis: in the setting of LV hypertrophy the presence of a typical pattern of midmyocardial late gadolinium enhancement in the basal to mid inferolateral wall may aid in differential diagnosis (10). Longitudinal strain has proven to be less useful in distinguishing AFD patients from other conditions associated with LV hypertrophy (14, 18, 50–52, 61, 75–80). Loss of base to apex gradient of LV circumferential strain, irrespectively of the increase in LV wall thickness, seems to be specific for AFD (12, 33, 34). Table 1 summarizes myocardial strain characteristics of AFD and of other forms of cardiac hypertrophy such as nonobstructive hypertrophic cardiomyopathy and cardiac amyloidosis.

**Table 1:** Strain echocardiography features of Anderson–Fabry disease and other conditions of left ventricular hypertrophy in adults.

	Left ventricle	Left atrium	Right ventricle
Anderson–Fabry disease	Reduced longitudinal strain in the basal posterior-lateral wall; reduced GLS, inversely and independently associated with LV mass (12–17), impaired subepicardial longitudinal strain at multilayer strain analysis (35), loss of normal circumferential strain base-to-apex gradient (12, 33, 34), reduced radial strain (9, 36–38), reduced constructive work (40, 41).	Reduced left atrial strain/strain rate parameters (48–52)	Reduced longitudinal systolic right ventricle strain and right ventricle free wall strain (12, 58, 59, 61–63)
Hypertrophic cardiomyopathy	Reduced longitudinal, circumferential and radial strain (75) reduced constructive work (76).	Reduced phasic left atrial strain (50, 53)	Reduced longitudinal systolic right ventricle strain and right ventricle free wall strain (61)
Cardiac amyloidosis	Reduced longitudinal strain with relative apical sparing pattern (18) and increased EFSR (77); reduced radial strain (78), reduced global constructive work and work efficiency (41, 79).	Impairment of left atrial strain more severe than in AFD (51, 52).	Reduced longitudinal systolic right ventricle strain and right ventricle free wall strain with increased apical ratio (51, 80).

GLS, global longitudinal strain; LV, left ventricular; EFRS, ejection fraction longitudinal systolic strain ratio.

## Conclusions

Advanced cardiac imaging has played a crucial role in defining features of the unique cardiac involvement due to AFD. Strain imaging by cardiac ultrasound is involved in many aspects: the initial diagnostic suspicion of AFD in case of evidence of unexplained heart damage, the differential diagnosis with other cardiomyopathies, the early detection of heart involvement in patients with already diagnosed AFD, the decisions regarding the initiation of chaperone or enzyme replacement therapy. Further large studies are warranted to ascertain the prognosticator value of LV longitudinal strain in defining patient risk profile and monitoring evolution of AFD cardiomyopathy. Research should be prompted to verify whether and at what extent advanced echocardiography may provide insights into the impact of disease-specific therapy on the heart of AFD patients.
